# Provision of Endodontic Treatment in Dentistry amid COVID-19: A Systematic Review and Clinical Recommendations

**DOI:** 10.1155/2021/8963168

**Published:** 2021-12-03

**Authors:** Farooq Ahmad Chaudhary, Ayesha Fazal, Muhammad Mohsin Javaid, Muhammad Waqar Hussain, Ammar Ahmed Siddiqui, Mawra Hyder, Mohammad Khursheed Alam

**Affiliations:** ^1^Department of Community Dentistry, School of Dentistry, Shaheed Zulfiqar Ali Bhutto Medical University, Islamabad, Pakistan; ^2^Bakhtawar Amin Medical & Dental College, Multan, Pakistan; ^3^Department of Preventive Dentistry, College of Dentistry, University of Hail, Saudi Arabia; ^4^Department of Preventive Dental Science, College of Dentistry, Jouf University, Saudi Arabia

## Abstract

**Background:**

The risk of acquiring COVID-19 during a pandemic is a major concern among health care workers. Dental professionals being in close proximity to the patients had been exposed more than other health care workers. Hence, all the standard operating procedures (SOPs) are strictly advised to be followed.

**Methods:**

A detailed relevant literature search was conducted in international databases such as PubMed, Web of Science, and Science Direct, from January 2020 to November 2020. All the studies that provided recommendations regarding endodontic procedures during the COVID-19 pandemic were included, and those that were not in the English language, case reports, book chapters, and short communications were excluded in this review. In the end, only 6 articles were selected for the systematic review considering that complete information regarding the provision of dental care in the time of COVID-19 with diagnostic accuracy (STARD) was provided.

**Results:**

Endodontic treatments were restricted to only emergency dental procedures, and all other patients have advised medications and catered through teledentistry. Endodontic emergencies were advised to be carried out with minimal aerosol production procedures.

**Conclusion:**

Provision of endodontic care during COVID-19 restricted to only emergency dental procedures by strictly following standard operating procedures. A protocol for COVID-19 prevention was followed by all the dentists and the dental staff in the dental clinics.

## 1. Introduction

The COVID-19 pandemic had a serious adverse physical and psychosocial impact on health care workers (HCW) all over the world [1]. This pandemic had endorsed very challenging conditions for HCW to work with since they are the frontline warriors [2]. Dentists being working in close proximity with the patients' oral cavities are at greater risk of acquiring this infection [3] which directly affects their physical and psychological health [4–7]. The three most common routes of transmission of this virus are direct transmission (coughing or sneezing), contact transmission (transmission through mouth and eyes), and transmission through aerosols [8]. The oral cavity could be one of the first sites of coronavirus entry, as it is attached directly to the angiotensin-converting enzyme- (ACE-) 2 receptor present in a tongue, the floor of the mouth, saliva, and other parts of the oral cavity [9–11]. Due to the widespread disease [12], utilization of resources should be restricted to the necessity of use, especially during dental practice. Therefore, only necessary and emergency dental procedures should be carried out with proper training and under expert supervision. In this regard, guidelines issued by governments of different regions are to be followed for medical and dental practices, which allow restricted procedures to be carried out following strict protocols [13]. Saliva and blood are the main components for this viral spread; hence, procedures requiring minimal aerosol production are encouraged to be practiced [14]. Moreover, oral rinses like chlorhexidine at 0.12% to 0.20% are meant to alter the concentration of bacteria, viruses, and fungi residing in the oral cavity [15]. Irrespective of the dental procedures to be carried out, the dentists and the other staff should follow all the protocols to restrict the spread of infection. In this regard, personal protective equipment (PPE) including hair caps, protective eyewear, surgical masks, and surgical gowns along with footwear should be worn while performing any dental procedure [16]. If any defect is found in the PPE, it should be disposed of immediately. The use of N-95 masks in daily use should be practiced, since it shows greater reliability to control spread of infection than wearing common surgical masks [17]. During this COVID-19 pandemic, regional governments and authorities of many countries have offered various guidelines to be followed by dentists during dental practice [18]. The guidelines vary from area to area, depending on the spread of the pandemic in that specific area [19]. In this article, the recommendations for endodontic dental care during this COVID-19 pandemic are discussed which need to be implemented and practiced by the general dentists and endodontic population. Therefore, the aim of this paper is to lay out guidelines regarding general dental practices and clinical endodontic management during COVID-19, since dentists are the most vulnerable group to this deadly virus.

## 2. Methods

### 2.1. Search Strategy

A detailed relevant literature search was conducted in international databases such as PubMed, Web of Science, and Science Direct, from January 2020 to November 2020. Additional studies were searched by using gray literature through Google Scholar. Open access journals from last past five years were explored in the filters. MeSH words used for search purposes on PubMed included COVID-19, SARS-CoV 2, dentistry, dental care, endodontic emergencies, and endodontic treatment. Keywords used for searching relevant data from Web of Science and Science Direct included endodontic care, endodontic guidelines, COVID pandemic, precautionary measures, and personal protective equipment. The major Boolean terms used for search purposes were “AND” and “OR.”

### 2.2. Inclusion and Exclusion Criteria

All the studies that provided recommendations regarding endodontic procedures during the COVID-19 pandemic were included, and those that were not in the English language, case reports, book chapters, and short communications were excluded in this review.

### 2.3. Selection of Studies

The process of selection of studies was carried out in two rounds by the authors A.F. and F.A.C. In each round, in case of disagreement between the two primary investigators, the decision of the third investigator M.H. was considered. In the first round, the authors reviewed the titles/abstracts of the articles. Four hundred and ninety-one articles were retrieved after the removal of duplication from the above-mentioned databases. Then, the articles with incomplete information/considering comorbidities, case reports, case series, short communications, and letters to editors from the study were removed, and only 15 articles were included. In the second round, articles with full texts were considered, and finally, 6 articles were selected for this systematic review considering that complete information regarding the provision of dental care in the time of COVID-19 with diagnostic accuracy (STARD) was provided.

### 2.4. Data Abstraction

After the initial literature search, title pages and abstracts were studied to check the relevance of the concerned study. Afterward, the full texts of the included studies were accessed for detailed review.

## 3. Results

### 3.1. Quality Assessment

The quality assessment to check the risk of bias for the two observational studies was performed by two investigators (A.F and F.A.C), independently which is shown in Table 1. It was done using a 9-item questionnaire designed by Hoy et al., which primarily measured the risk of bias. The included observational studies were marked according to nine questions by the investigators (A.F and F.A.C.), and an average of two values was considered the final answer for each question. Low risk of bias was considered if the score was between 0 and 3, while scores of the medium and high risk of bias were between 4 and 6 and 7 and 9, respectively [20].

The quality assessment of review articles was measured using the quality assessment form for multiple systematic reviews (AMSTAR) by two investigators (A.F. and M.M.J.), shown in Table 2. In case of disagreement between the two primary investigators, the third investigator (F.A.C.) was considered to make the decision. The review articles were marked as yes (Y), no (N), cannot answer (CA), and not applicable (NA) [21].

**Figure 1:**
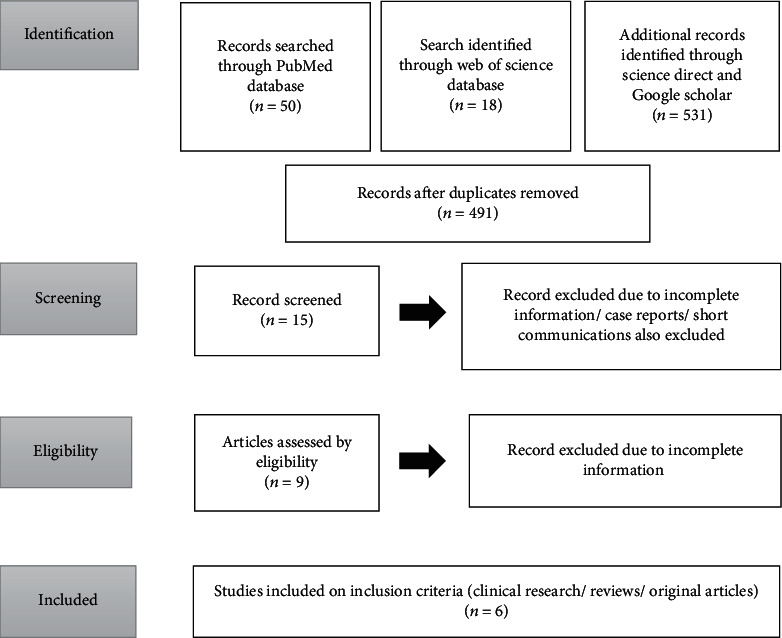
PRISMA flow diagram showing study characteristics.

Six studies were included, considering clinical research, literature and systematic reviews, and original articles. The research publications were included based on the flow diagram following 2009 guidelines which is shown in Figure 1.

Palliative care for restriction of aerosol production is a successful endodontic treatment option during this COVID-19 pandemic (Table 2). Patel et al. revealed that apart from treatment issues, delay in COVID-19 testing or treatment due to perceived risk of COVID-19 infection could result in failure of tooth [22]. Four-handed dentistry is found to be useful with the use of a rubber dam to minimize aerosol production. Care should be taken at patients' as well as dentists' ends. At the dentists' end, they should always wear personal protective equipment and face shields [23], whereas at the patients' end, they should visit the dental clinic alone, with all the necessary precautionary measures, and only one or two patients are allowed to enter the clinic at one time, depending on the space availability. Before treatment, the appointment should be planned to avoid crowding in the dental clinic and should wait outside the clinic in their transport for their turn.

Cosmetic/nonurgent treatments should be delayed, and treatments for dental emergencies like symptomatic irreversible pulpitis should be performed [24]. During the treatment, screening of patients is to be done as per standard operational procedures. Hand hygiene with alcohol-based hand rub with 60-75% alcohol should be done at the start of the clinic. Use soap and water for 20 seconds if hands are visibly soiled, and it is recommended to gargle with 0.5-1% hydrogen peroxide before the start of treatment. Use of extraoral radiographs instead of intraoral radiographs and use of rubber dams and saliva ejectors should be made mandatory. Avoid using handpieces, ultrasonic instruments, and air-water syringes. Resorbable sutures should be placed to avoid unnecessary appointments of the patients. At the end of the treatment, proper cleaning and disinfection of the dental office should be performed, followed by cleaning of personal protective equipment (PPE) with soap and water [23].

Ates et al. compared dental practices of endodontists and general dental practitioners (GDPs) and reported better results for general dental practitioners (70.7%) as compared to endodontists (66.9%) regarding initial screening before coming to the dental clinic. Similarly, more GDPs (72%) monitored the temperature of the patients at arrival as compared to endodontists (62.5%), and antibiotics and analgesics have been prescribed more by the GDPs as compared to endodontists. However, rubber dams were more commonly used by endodontists (35.5%) than general dental practitioners (27.1%). Pulpectomy was found more common in practice by endodontists (61%), and pulpotomy was found to be done more by GDPs (41%) [25].

**Figure 2:**
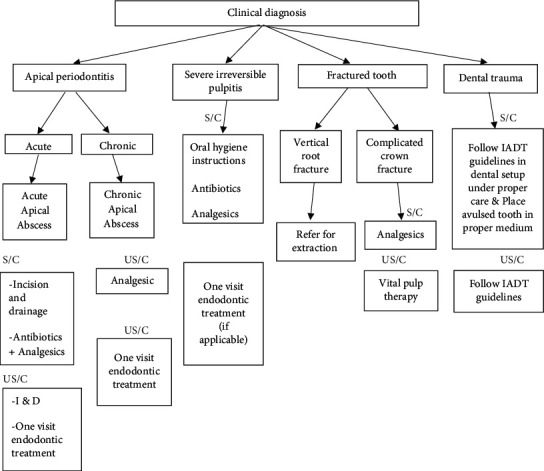
Endodontic clinical recommendations amid the COVID-19 pandemic.

Abramovitz et al. explained operatory considerations during the COVID-19 pandemic considering that only symptomatic patients were to be treated according to the main complaint [26]. The clinical diagnosis regarding various treatments is recommended in Figure 2.

During the current COVID-19 pandemic, extra care should be taken by all the dentists and dental staff, as well as by the patients. Personal protective equipment and face shields should be made compulsory for all dental staff, and the “one patient one attendant” policy should be followed. The patients should avoid coming to clinics/hospitals with minor complaints and only visit clinics in case of dental emergencies. Appointments should be planned before going to the clinic, to decrease the waiting time and avoid overlapping of patients causing overcrowding in the clinics. Procedures involving minimal aerosol production should be performed only. Regarding compliance with the precautionary measures during this pandemic, general dentists showed better results as compared to an endodontist.  Table 3 presents all the studies included in this review, with a description of the outcome of each paper.

## 4. Discussion

The COVID-19 pandemic has led to changes in the treatment dynamics of the health care system, especially in dentistry. Due to this situation, only dental emergencies were allowed by the concerned authorities to minimize the chances of the spread of coronavirus through dentistry [25, 26]. Appointments of patients should be scheduled in balance with the overall workload, avoiding overcrowding with all the necessary precautionary measures like face masks and hand washing [28]. This finding is relevant to the study of the influenza outbreak in 2009, where hand washing and wearing face masks among the dentists and dental students were advised [29]. Similarly, self-care practices including frequent hand washing, avoiding crowded places, and improvement in dietary intakes were practiced by the dentists amid the swine flu outbreak in 2009 [30]. The COVID-19 vaccination drive is carried out in many countries, and the effectiveness of this vaccine depends on its coverage because herd immunity will only develop if the vaccination rate is high in the population [31]. Therefore, till the population reached that stage of herd immunity, all the precautions and necessary protocols need to be followed by health professionals including dentists. Various pharmaceutical companies have developed the vaccine for COVID-19; however, their effectiveness towards new strains of the COVID-19 virus is still under trial [32]. In this regard, various vaccine trials are undergoing in China, Russia, and America, and no associated major side effect was reported with the use of these vaccines, except slight pain on the site of injection and discomfort [31]. Similar findings of pain on the site of injection and lethargy were reported during influenza vaccine trials among the dentistry staff and students in Malaysia [33]. The dental guidelines need to be continuously formulated and amended in accordance with the discoveries of the new strains of COVID-19 viruses in various regions of the world [34]. In contrary to this, albeit the awareness campaigns through social media and websites were conducted during the swine-origin influenza outbreak, no dental guidelines were issued by WHO or the concerned authorities [35].

Endodontic procedures are some of the most commonly practiced dental procedures [36, 37]. Pajpani et al. investigated the rapid response to dental care in hospital-based dental care and reported that dentoalveolar procedures were the most prevalent (84%) among all the procedures. Aerosol and nonaerosol-generating procedures were carried out [38]. In another study by Obeidat et al. in Jordan, they reported a ninety percent reduction in outpatient flow and mostly the patients who showed up were having endodontic cases (51.9%) [39]. Similar findings were noted during the Ebola virus outbreak when the dental procedures were restricted to emergency dental care only [40].

New challenges require new solutions; hence, the dental services need to evolve during the current pandemic situation along with the involvement of the dental practices in the communities and not just being restricted to the dental clinics [41]. Gianluca et al. stated that new techniques have been introduced in endodontic practices to improve the efficiency of the outcomes [42]. Moreover, it is advised to refer the suspected cases to the facilities equipped with airborne isolation transmission through aerosols (AIIRS) [43]. Albeit the status of fever is being questioned from the outdoor patients, a history of diarrhea should also be asked. Hand washing and wearing of masks should be made compulsory till the pandemic is completely over [44].

Various techniques to overcome the pandemic situation have been introduced and implemented worldwide, including work from home, virtual teamwork, and virtual leadership and management [45]. In dentistry, teledentistry has been practiced to overcome this situation [46]. However, dental emergencies had to be catered according to the standard operating procedures during this pandemic situation.

This review has some limitations. The articles in the English language were included only, leading to selection bias. Although registering the protocols of the systematic review is not mandated by medical journal editors [47], they are highly recommended and the review protocol of this systematic review is not registered. We used limited databases to search for studies for this review which may lead to exclusion of some studies which are not indexed by these databases.

## 5. Recommendations

Provision of endodontic care during COVID-19 had to be restricted to emergency dental procedures, following necessary standard operating procedures. Otherwise, patients were advised to have medications and the problems were catered through teledentistry. All the reported protocols for COVID-19 prevention should be followed by the dentists and the dental staff in the dental clinics. The provision of endodontic treatments is made with respect to guidelines explained in Figure 2, and screened and unscreened patients need to be treated according to these guidelines.

## Figures and Tables

**Table 1:** Quality assessment of included studies (risk of bias).

Risks of bias items	Patel et al. (2020)	Ates et al. (2020)
Was the study's target population a close representation of the national population in relation to relevant variables, e.g., age, sex, and occupation?	1	1
Was the sampling frame a true or close representation of the target population?	1	1
Was some form of random selection used to select the sample?	0	1
Was the likelihood of nonresponse bias minimal?	1	1
Were data collected directly from the subjects (as opposed to a proxy)?	1	1
Was an acceptable case definition used in the study?	1	1
Was the study instrument that measured the parameter of interest shown to have reliability and validity	1	0
Were the numerator(s) and denominator(s) for the parameter of interest appropriate	0	0
Was the same mode of data collection used for all subjects?	1	1
Total points	7 High	7 High

Summary on the overall risk of study bias: low risk = 0-3, medium risk = 4-6, and high risk = 7-9. 0 = yes, low risk; 1 = no, high risk.

**Table 2:** Quality assessment form for multiple systematic reviews (AMSTAR).

	Silva et al. (2020)	Azim et al. (2020)	Banakar et al. (2020)	Abramovitz et al. (2020)
1. Was a priori design provided?	Y	Y	Y	Y
2. Were there duplicate study selection and data extraction?	N	N	Y	N
3. Was a comprehensive literature search performed?	Y	Y	Y	N
4. Was the status of publication (i.e., grey literature) used as an inclusion criterion?	Y	Y	Y	N
5. Was a list of studies (included and excluded) provided?	N	Y	Y	N
6. Were the characteristics of the included studies provided?	N	Y	Y	N
7. Was the scientific quality of the included studies assessed and documented?	N	N	N	Y
8. Was the scientific quality of the included studies used appropriately in formulating conclusions?	N	N	Y	Y
9. Were the methods used to combine the findings of studies appropriate?	Y	Y	Y	Y
10. Was the likelihood of publication bias assessed?	Y	N	N	N
11. Was the conflict of interest included?	N	Y	Y	Y

Y: yes; N: no; CA: cannot answer; NA: not applicable.

**Table 3:** An overview of recommendations on endodontic interventions amid the COVID-19 pandemic.

S. no.	Title	Type of study	Outcome of paper	Author (year/country)
1	To drill or not to drill: management of endodontic emergencies and in-process patients during the COVID-19 pandemic	Clinical research	(i) Out of 21 patients (with complaints of 25 teeth) at a follow-up rate of 96%, 83% received palliative care and required no further endodontic treatment (ii) With 31 teeth receiving partial/full root canal treatment in a mean time of 13 weeks, at a recall rate of 100%, 77% did not show any adverse effects of the delay	Patel et al. (March 2020/Texas, USA) [22]
2	Recommendations for managing endodontic emergencies during the coronavirus disease 2019 outbreak	Review article	(i) Minimal aerosol production procedures, with 4-handed dentistry, are recommended (ii) Use of preprocedural antiseptic mouth rinses, rubber dam, and sterile burs for access preparation (iii) Dentists should wear personal protective equipment with face shields	Silva et al. (May 2020/USA) [23]
3	Clinical endodontic management during the COVID-19 pandemic: a literature review and clinical recommendations	Review article	(i) If flu-like symptoms persist/or the patient came in contact with the positive case, then defer the treatment (ii) The patient should come alone and gargle with 0.5-1% hydrogen peroxide before initiating treatment	Azim et al. (2020/New York, USA) [24]
4	COVID-19 transmission risk and protective protocols in dentistry: a systematic review	Systematic review	(i) Before, during, and after the treatment, guidelines are to be followed as per standard operating procedures (ii) Patients to be appointed as per space in the waiting area (iii) Defer treatment of nonurgent procedures (iv) Proper disinfection of the clinic along with soap and water	Banakar et al. (2020/Iran) [27]
5	Differences in endodontic management by endodontists and general dental practitioners in COVID-19 times	Original article	(i) Initial screening was practiced better by the general dental practitioners (GDPs) as compared to endodontists (ii) The use of rubber dam and pulpectomy procedures were practiced more by endodontists (iii) Use of antibiotics/analgesics and pulpotomy procedures were followed by GDPs more as compared to endodontists	Ates et al. (2020/Jordan) [25]
6	Dental care during the coronavirus disease 2019 (COVID-19) outbreak: operatory considerations and clinical aspects	Review article	(i) Treatment of asymptomatic patients was to be delayed (ii) Symptomatic treatment of patients was to be carried out depending on the main complaint, minimizing the procedure time and following proper standard operating procedures	Abramovitz et al. (2020/USA) [26]

## Data Availability

All data are available within the manuscript.

## Abbreviations

ACE: Angiotensin-converting enzymes
PPE: Personal protective equipment
COVID-19: Coronavirus disease 2019
GDPs: General dental practitioners.

## Supplementary Materials

Supplementary Materials We have uploaded files as supplementary material. The file is the PRISMA checklist that was used as a guideline to carry out the systematic review. This systematic review was performed following the PRISMA guidelines.
